# Crystal structure of 3,4-di­meth­oxy­phenol

**DOI:** 10.1107/S2056989015022860

**Published:** 2015-12-06

**Authors:** Heather A. Mills-Robles, Vasumathi Desikan, James A. Golen, David R. Manke

**Affiliations:** a Department of Science & Math, Massasoit Community College, 1 Massasoit Boulevard, Brockton, MA 02302, USA; b Department of Chemistry and Biochemistry, University of Massachusetts Dartmouth, 285 Old Westport Road, North Dartmouth, MA 02747, USA

**Keywords:** crystal structure, hydrogen bonding, phenols

## Abstract

The title compound, C_8_H_10_O_3_, has two planar mol­ecules in the asymmetric unit possessing mean deviations from planarity of 0.051 and 0.071 Å. In the crystal, there are two distinct infinite chains, both along [010]. The chains are formed by O—H⋯O inter­actions between the phenol and both the 3-meth­oxy and the 4-meth­oxy groups.

## Related literature   

For the crystal structure of the related 4-[(2,3-di­methyl­but-3-en-2-yl)­oxy]-3-meth­oxy­phenol, see: Yamamoto *et al.* (2014 ▸). For the crystal structure of 3,4,5-tri­meth­oxy­phenol, see: Jia *et al.* (2012 ▸). For background and crystal structures solved during the study, see: McDonald *et al.* (2015 ▸); Nguyen *et al.* (2015 ▸).
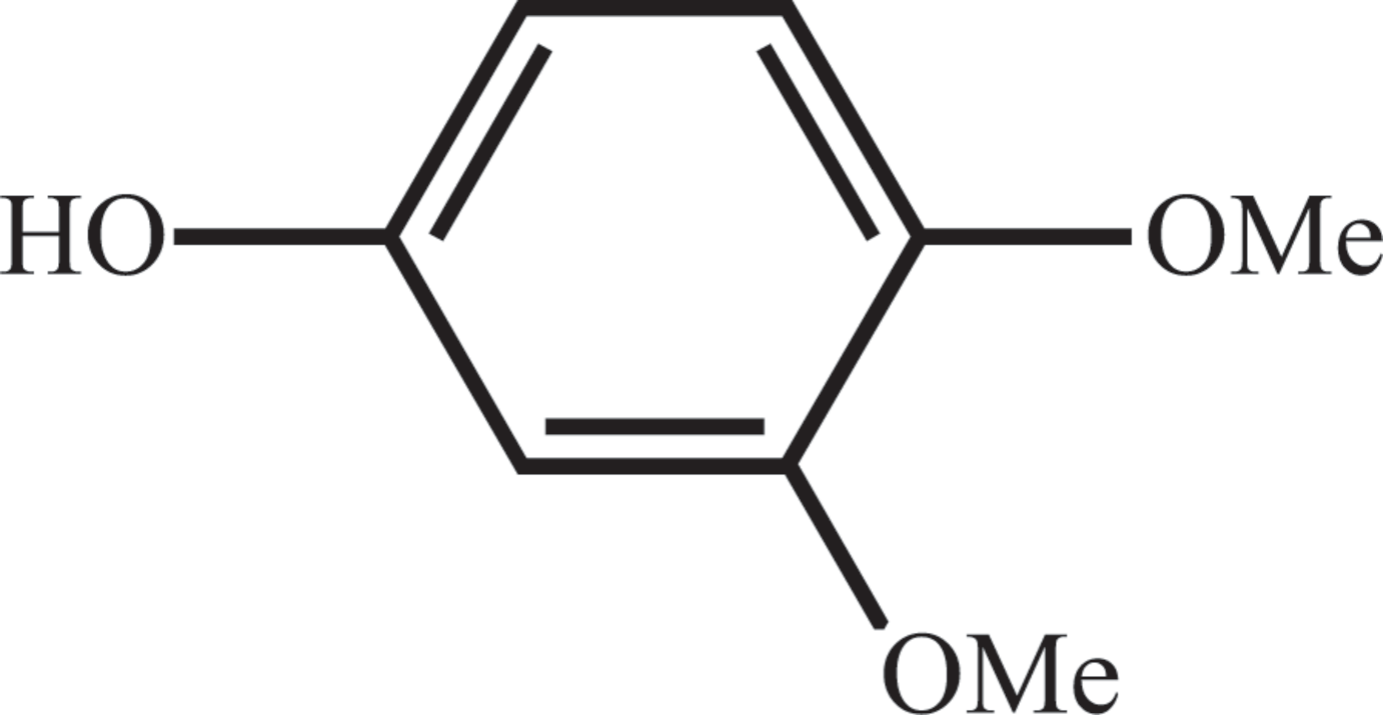


## Experimental   

### Crystal data  

C_8_H_10_O_3_
*M* *_r_* = 154.16
Orthorhombic, 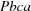

*a* = 8.7477 (4) Å
*b* = 13.8218 (7) Å
*c* = 26.6422 (13) Å
*V* = 3221.3 (3) Å^3^
*Z* = 16
Mo *K*α radiation
μ = 0.10 mm^−1^
*T* = 120 K
0.5 × 0.4 × 0.4 mm

### Data collection  

Bruker Venture D8 CMOS diffractometer
Absorption correction: multi-scan (*SADABS*; Bruker, 2014 ▸) *T*_min_ = 0.700, *T*_max_ = 0.746
29914 measured reflections
3996 independent reflections
3360 reflections with *I* > 2σ(*I*)
*R*_int_ = 0.032

### Refinement  

*R*[*F* ^2^ > 2σ(*F* ^2^)] = 0.042
*wR*(*F* ^2^) = 0.116
*S* = 1.04
3996 reflections
205 parameters
2 restraints
H atoms treated by a mixture of independent and constrained refinement
Δρ_max_ = 0.25 e Å^−3^
Δρ_min_ = −0.25 e Å^−3^

### 

Data collection: *APEX2* (Bruker, 2014 ▸); cell refinement: *SAINT* (Bruker, 2014 ▸); data reduction: *SAINT*; program(s) used to solve structure: *SHELXS97* (Sheldrick, 2008 ▸); program(s) used to refine structure: *SHELXL2014* (Sheldrick, 2015 ▸) and *olex2.refine* (Bourhis *et al.*, 2015 ▸); molecular graphics: *OLEX2* (Dolomanov *et al.*, 2009 ▸); software used to prepare material for publication: *OLEX2* and *publCIF* (Westrip, 2010 ▸).

## Supplementary Material

Crystal structure: contains datablock(s) I. DOI: 10.1107/S2056989015022860/ff2145sup1.cif

Structure factors: contains datablock(s) I. DOI: 10.1107/S2056989015022860/ff2145Isup2.hkl

Supporting information file. DOI: 10.1107/S2056989015022860/ff2145Isup3.cml

**Fig. 1.**
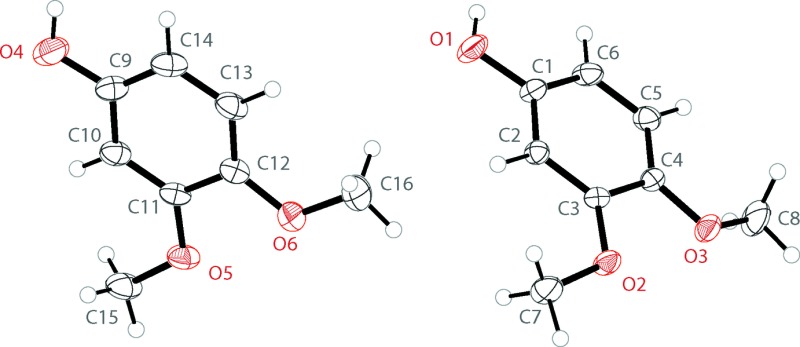
Molecular structure of the title compound, showing the atom-labelling scheme. Displacement ellipsoids are drawn at the 50% probability level. H atoms are drawn as spheres of arbitrary radius.

. DOI: 10.1107/S2056989015022860/ff2145fig1.tif
Mol­ecular structure of the title compound, showing the atom-labelling scheme. Displacement ellipsoids are drawn at the 50% probability level. H atoms are drawn as spheres of arbitrary radius.

**Fig. 2.**
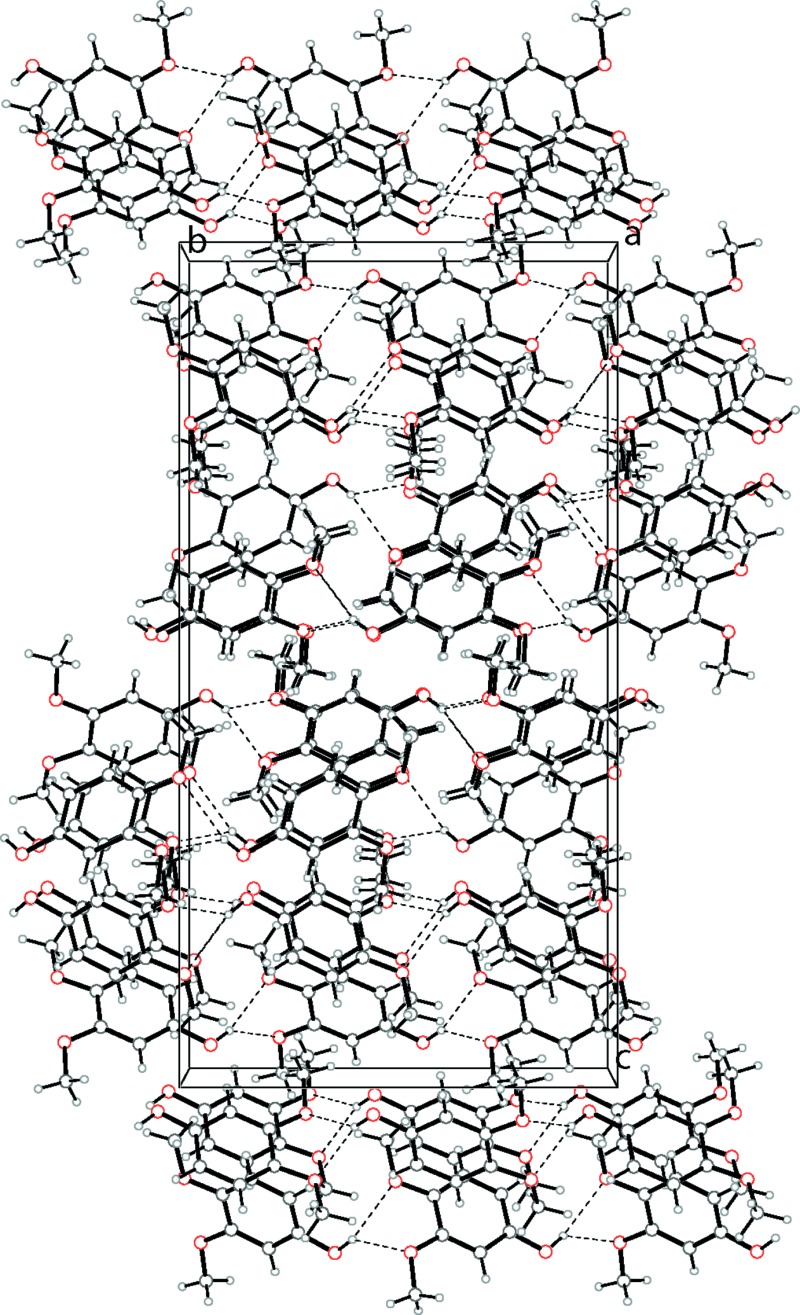
Molecular packing of the title compound with hydrogen bonding shown as dashed lines.

. DOI: 10.1107/S2056989015022860/ff2145fig2.tif
Mol­ecular packing of the title compound with hydrogen bonding shown as dashed lines.

CCDC reference: 1439495

Additional supporting information:  crystallographic information; 3D view; checkCIF report

## Figures and Tables

**Table 1:** Hydrogen-bond geometry (Å, °)

*D*—H⋯*A*	*D*—H	H⋯*A*	*D*⋯*A*	*D*—H⋯*A*
O1—H1⋯O2^i^	0.86 (1)	2.25 (1)	2.9663 (12)	141 (2)
O1—H1⋯O3^i^	0.86 (1)	2.13 (1)	2.8834 (13)	145 (2)
O4—H4⋯O5^i^	0.86 (1)	2.15 (2)	2.8384 (13)	137 (2)
O4—H4⋯O6^i^	0.86 (1)	2.37 (1)	3.1107 (14)	145 (2)

Symmetry code: (i) 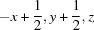.

## supplementary crystallographic information

### S1. Comment

In a continuing collaborative study of the solid state structure of aromatic alcohols between UMass Dartmouth and Massasoit Community College (McDonald *et al.*, 2015; Nguyen *et al.*, 2015), we report herein the structure of 3,4-di­meth­oxy­phenol. A similar 3,4-di­alk­oxy­phenol complex has been structurally characterized (Yamamoto *et al.*, 2014) and demonstrates tip-to-tail hydrogen bonding with the 4-alk­oxy group. The structure of the tris­ubstituted 3,4,5-tri­meth­oxy­phenol demonstrates a similar inter­action, with just the 4-meth­oxy group involved in hydrogen bonding (Jia *et al.*, 2012). In contrast, the title compound exhibits hydrogen bonding chains with inter­actions involving the meth­oxy groups at both the 3 and 4 positions.

The molecular structure of the title compound is shown in Figure 1. There are two molecules in the asymmetric unit, with non-hydrogen atoms possessing mean deviations from the plane of 0.051 Å and 0.071 Å. There are two distinct hydrogen bonding chains which both propagate along [010]. One is formed by O1–H1···O2 and O1–H1···O3 inter­actions, and the other by O4–H4···O5 and O4–H4···O6 inter­actions. The packing of the title compound indicating hydrogen bonding is shown in Figure 2.

### S2. Experimental

A commercial sample (Aldrich) was used for crystallization. Single crystals suitable for X-ray diffraction studies were grown by slow evaporation of a methyl­ene chloride solution.

### S3. Refinement

All non-hydrogen atoms were refined anisotropically (Olex2) by full matrix least squares on F^2^. Hydrogen atoms H1 and H4 were found from a Fourier difference map, and refined with a fixed distance of 0.86 (0.005) Å and isotropic displacement parameters of 1.50 times *U*_eq_ of the parent O atoms. The remaining hydrogen atoms were placed in calculated positions and then refined with a riding model with C–H lengths of 0.95 Å (*sp*^2^) and 0.98 Å (*sp*^3^) with isotropic displacement parameters set to 1.20 (*sp*^2^) and 1.50 (*sp*^3^) times *U*_eq_ of the parent C atom.

### Crystal data

**Table:**

C_8_H_10_O_3_	*F*(000) = 1312
*M**_r_* = 154.16	*D*_x_ = 1.271 Mg m^−^^3^
Orthorhombic, *P**b**c**a*	Mo *K*α radiation, λ = 0.71073 Å
Hall symbol: -P 2ac 2ab	Cell parameters from 9890 reflections
*a* = 8.7477 (4) Å	θ = 3.0–28.3°
*b* = 13.8218 (7) Å	µ = 0.10 mm^−^^1^
*c* = 26.6422 (13) Å	*T* = 120 K
*V* = 3221.3 (3) Å^3^	Block, brown
*Z* = 16	0.5 × 0.4 × 0.4 mm

### Data collection

**Table:**

Bruker Venture D8 CMOS diffractometer	3996 independent reflections
Radiation source: Mo	3360 reflections with *I* > 2σ(*I*)
TRIUMPH monochromator	*R*_int_ = 0.032
φ and ω scans	θ_max_ = 28.4°, θ_min_ = 2.9°
Absorption correction: multi-scan (*SADABS*; Bruker, 2014)	*h* = −11→10
*T*_min_ = 0.700, *T*_max_ = 0.746	*k* = −18→18
29914 measured reflections	*l* = −35→34

### Refinement

**Table:**

Refinement on *F*^2^	2 restraints
Least-squares matrix: full	Hydrogen site location: mixed
*R*[*F*^2^ > 2σ(*F*^2^)] = 0.042	H atoms treated by a mixture of independent and constrained refinement
*wR*(*F*^2^) = 0.116	*w* = 1/[σ^2^(*F*_o_^2^) + (0.0552*P*)^2^ + 1.1413*P*] where *P* = (*F*_o_^2^ + 2*F*_c_^2^)/3
*S* = 1.04	(Δ/σ)_max_ = 0.001
3996 reflections	Δρ_max_ = 0.25 e Å^−^^3^
205 parameters	Δρ_min_ = −0.25 e Å^−^^3^

### Special details

**Table:**

Experimental. Absorption correction: *SADABS2014*/4 (Bruker,2014/4) was used for absorption correction. wR2(int) was 0.0791 before and 0.0531 after correction. The Ratio of minimum to maximum transmission is 0.9391. The λ/2 correction factor is 0.00150.
Geometry. All e.s.d.'s (except the e.s.d. in the dihedral angle between two l.s. planes) are estimated using the full covariance matrix. The cell e.s.d.'s are taken into account individually in the estimation of e.s.d.'s in distances, angles and torsion angles; correlations between e.s.d.'s in cell parameters are only used when they are defined by crystal symmetry. An approximate (isotropic) treatment of cell e.s.d.'s is used for estimating e.s.d.'s involving l.s. planes.

### Fractional atomic coordinates and isotropic or equivalent isotropic displacement parameters (Å^2^)

**Table:**

	*x*	*y*	*z*	*U*_iso_*/*U*_eq_	
O1	0.31642 (12)	0.55792 (6)	0.46341 (4)	0.0364 (2)	
H1	0.2656 (18)	0.6010 (10)	0.4472 (6)	0.055*	
O2	0.41171 (10)	0.21648 (5)	0.45901 (3)	0.0274 (2)	
O3	0.23732 (11)	0.19341 (6)	0.38304 (3)	0.0322 (2)	
C1	0.29128 (13)	0.46989 (8)	0.44100 (4)	0.0244 (2)	
C2	0.36772 (12)	0.39078 (7)	0.46188 (4)	0.0215 (2)	
H2	0.4337	0.3994	0.4899	0.026*	
C3	0.34642 (12)	0.29982 (7)	0.44142 (4)	0.0198 (2)	
C4	0.25063 (13)	0.28729 (8)	0.39961 (4)	0.0224 (2)	
C5	0.17669 (14)	0.36617 (8)	0.37932 (4)	0.0267 (2)	
H5	0.1123	0.3579	0.3509	0.032*	
C6	0.19567 (14)	0.45826 (8)	0.40017 (5)	0.0280 (3)	
H6	0.1433	0.5123	0.3864	0.034*	
C7	0.49570 (17)	0.22270 (9)	0.50473 (5)	0.0374 (3)	
H7A	0.5365	0.1588	0.5133	0.056*	
H7B	0.5803	0.2685	0.5006	0.056*	
H7C	0.4280	0.2451	0.5317	0.056*	
C8	0.1208 (2)	0.17531 (11)	0.34658 (6)	0.0538 (5)	
H8A	0.1214	0.1066	0.3375	0.081*	
H8B	0.0209	0.1924	0.3607	0.081*	
H8C	0.1402	0.2145	0.3166	0.081*	
O4	0.27270 (14)	0.84893 (7)	0.72098 (4)	0.0463 (3)	
H4	0.319 (2)	0.8911 (12)	0.7029 (7)	0.069*	
O5	0.06921 (10)	0.52789 (6)	0.71498 (3)	0.0313 (2)	
O6	0.20065 (12)	0.49211 (7)	0.63132 (3)	0.0370 (2)	
C9	0.26184 (15)	0.76288 (9)	0.69618 (5)	0.0313 (3)	
C10	0.17021 (14)	0.69220 (9)	0.71881 (5)	0.0288 (3)	
H10	0.1198	0.7055	0.7496	0.035*	
C11	0.15354 (13)	0.60291 (8)	0.69608 (4)	0.0250 (2)	
C12	0.22572 (14)	0.58329 (9)	0.65010 (4)	0.0278 (3)	
C13	0.31638 (15)	0.65389 (10)	0.62848 (5)	0.0337 (3)	
H13	0.3660	0.6411	0.5975	0.040*	
C14	0.33589 (15)	0.74365 (10)	0.65161 (5)	0.0346 (3)	
H14	0.3998	0.7912	0.6367	0.042*	
C15	0.01260 (18)	0.53863 (10)	0.76497 (5)	0.0403 (3)	
H15A	−0.0456	0.4808	0.7744	0.060*	
H15B	0.0986	0.5469	0.7881	0.060*	
H15C	−0.0540	0.5955	0.7667	0.060*	
C16	0.2624 (2)	0.47148 (13)	0.58306 (6)	0.0560 (5)	
H16A	0.2374	0.4048	0.5737	0.084*	
H16B	0.2187	0.5161	0.5583	0.084*	
H16C	0.3737	0.4794	0.5839	0.084*	

### Atomic displacement parameters (Å^2^)

**Table:**

	*U*^11^	*U*^22^	*U*^33^	*U*^12^	*U*^13^	*U*^23^
O1	0.0473 (6)	0.0169 (4)	0.0450 (5)	0.0030 (4)	−0.0158 (4)	−0.0031 (4)
O2	0.0317 (4)	0.0177 (4)	0.0326 (4)	0.0028 (3)	−0.0104 (3)	0.0003 (3)
O3	0.0436 (5)	0.0208 (4)	0.0322 (4)	−0.0027 (4)	−0.0134 (4)	−0.0036 (3)
C1	0.0267 (5)	0.0170 (5)	0.0295 (6)	−0.0015 (4)	−0.0012 (4)	0.0002 (4)
C2	0.0210 (5)	0.0201 (5)	0.0234 (5)	−0.0016 (4)	−0.0024 (4)	0.0003 (4)
C3	0.0189 (5)	0.0179 (5)	0.0227 (5)	0.0003 (4)	0.0007 (4)	0.0030 (4)
C4	0.0248 (5)	0.0195 (5)	0.0229 (5)	−0.0038 (4)	−0.0008 (4)	0.0002 (4)
C5	0.0282 (6)	0.0260 (5)	0.0260 (5)	−0.0040 (5)	−0.0078 (4)	0.0042 (4)
C6	0.0292 (6)	0.0212 (5)	0.0337 (6)	0.0008 (4)	−0.0067 (5)	0.0070 (4)
C7	0.0450 (7)	0.0256 (6)	0.0416 (7)	0.0050 (5)	−0.0209 (6)	0.0027 (5)
C8	0.0796 (12)	0.0334 (7)	0.0484 (9)	−0.0055 (8)	−0.0363 (8)	−0.0082 (6)
O4	0.0618 (7)	0.0258 (5)	0.0513 (6)	−0.0033 (5)	0.0108 (5)	0.0018 (4)
O5	0.0317 (4)	0.0287 (4)	0.0335 (5)	−0.0021 (3)	0.0081 (4)	0.0079 (3)
O6	0.0491 (6)	0.0340 (5)	0.0279 (4)	−0.0052 (4)	0.0066 (4)	−0.0012 (4)
C9	0.0318 (6)	0.0236 (5)	0.0385 (6)	0.0032 (5)	0.0006 (5)	0.0063 (5)
C10	0.0289 (6)	0.0269 (6)	0.0306 (6)	0.0062 (5)	0.0052 (5)	0.0065 (4)
C11	0.0207 (5)	0.0261 (5)	0.0283 (6)	0.0021 (4)	0.0008 (4)	0.0097 (4)
C12	0.0277 (6)	0.0288 (6)	0.0269 (6)	0.0013 (5)	0.0000 (4)	0.0056 (5)
C13	0.0346 (7)	0.0366 (6)	0.0300 (6)	0.0012 (5)	0.0094 (5)	0.0081 (5)
C14	0.0313 (6)	0.0311 (6)	0.0413 (7)	−0.0012 (5)	0.0077 (5)	0.0124 (5)
C15	0.0448 (8)	0.0339 (6)	0.0421 (7)	0.0050 (6)	0.0214 (6)	0.0106 (6)
C16	0.0853 (13)	0.0480 (9)	0.0348 (7)	−0.0094 (9)	0.0195 (8)	−0.0073 (7)

### Geometric parameters (Å, º)

**Table:**

O1—H1	0.859 (5)	O4—H4	0.860 (5)
O1—C1	1.3731 (13)	O4—C9	1.3638 (16)
O2—C3	1.3684 (12)	O5—C11	1.3686 (14)
O2—C7	1.4251 (14)	O5—C15	1.4285 (15)
O3—C4	1.3754 (13)	O6—C12	1.3735 (15)
O3—C8	1.4302 (16)	O6—C16	1.4236 (17)
C1—C2	1.3971 (15)	C9—C10	1.4002 (17)
C1—C6	1.3816 (16)	C9—C14	1.3786 (18)
C2—H2	0.9500	C10—H10	0.9500
C2—C3	1.3829 (14)	C10—C11	1.3824 (17)
C3—C4	1.4048 (15)	C11—C12	1.4045 (16)
C4—C5	1.3781 (16)	C12—C13	1.3831 (17)
C5—H5	0.9500	C13—H13	0.9500
C5—C6	1.3986 (16)	C13—C14	1.3957 (19)
C6—H6	0.9500	C14—H14	0.9500
C7—H7A	0.9800	C15—H15A	0.9800
C7—H7B	0.9800	C15—H15B	0.9800
C7—H7C	0.9800	C15—H15C	0.9800
C8—H8A	0.9800	C16—H16A	0.9800
C8—H8B	0.9800	C16—H16B	0.9800
C8—H8C	0.9800	C16—H16C	0.9800
			
C1—O1—H1	108.2 (13)	C9—O4—H4	110.7 (14)
C3—O2—C7	117.19 (9)	C11—O5—C15	116.82 (10)
C4—O3—C8	116.31 (10)	C12—O6—C16	116.88 (11)
O1—C1—C2	116.34 (10)	O4—C9—C10	116.09 (11)
O1—C1—C6	122.85 (10)	O4—C9—C14	123.55 (12)
C6—C1—C2	120.81 (10)	C14—C9—C10	120.35 (12)
C1—C2—H2	120.3	C9—C10—H10	120.2
C3—C2—C1	119.35 (10)	C11—C10—C9	119.65 (11)
C3—C2—H2	120.3	C11—C10—H10	120.2
O2—C3—C2	125.04 (9)	O5—C11—C10	124.89 (11)
O2—C3—C4	114.63 (9)	O5—C11—C12	114.65 (10)
C2—C3—C4	120.33 (9)	C10—C11—C12	120.47 (11)
O3—C4—C3	114.92 (9)	O6—C12—C11	115.02 (10)
O3—C4—C5	125.51 (10)	O6—C12—C13	125.96 (11)
C5—C4—C3	119.56 (10)	C13—C12—C11	119.00 (11)
C4—C5—H5	119.7	C12—C13—H13	119.6
C4—C5—C6	120.57 (10)	C12—C13—C14	120.89 (12)
C6—C5—H5	119.7	C14—C13—H13	119.6
C1—C6—C5	119.37 (10)	C9—C14—C13	119.62 (11)
C1—C6—H6	120.3	C9—C14—H14	120.2
C5—C6—H6	120.3	C13—C14—H14	120.2
O2—C7—H7A	109.5	O5—C15—H15A	109.5
O2—C7—H7B	109.5	O5—C15—H15B	109.5
O2—C7—H7C	109.5	O5—C15—H15C	109.5
H7A—C7—H7B	109.5	H15A—C15—H15B	109.5
H7A—C7—H7C	109.5	H15A—C15—H15C	109.5
H7B—C7—H7C	109.5	H15B—C15—H15C	109.5
O3—C8—H8A	109.5	O6—C16—H16A	109.5
O3—C8—H8B	109.5	O6—C16—H16B	109.5
O3—C8—H8C	109.5	O6—C16—H16C	109.5
H8A—C8—H8B	109.5	H16A—C16—H16B	109.5
H8A—C8—H8C	109.5	H16A—C16—H16C	109.5
H8B—C8—H8C	109.5	H16B—C16—H16C	109.5
			
O1—C1—C2—C3	179.40 (10)	O4—C9—C10—C11	−179.88 (11)
O1—C1—C6—C5	179.69 (11)	O4—C9—C14—C13	−179.08 (12)
O2—C3—C4—O3	0.15 (14)	O5—C11—C12—O6	−0.06 (15)
O2—C3—C4—C5	178.86 (10)	O5—C11—C12—C13	−178.59 (11)
O3—C4—C5—C6	178.24 (11)	O6—C12—C13—C14	−178.68 (12)
C1—C2—C3—O2	−178.48 (10)	C9—C10—C11—O5	178.92 (11)
C1—C2—C3—C4	0.94 (16)	C9—C10—C11—C12	−1.08 (17)
C2—C1—C6—C5	−0.61 (18)	C10—C9—C14—C13	1.42 (19)
C2—C3—C4—O3	−179.33 (10)	C10—C11—C12—O6	179.94 (11)
C2—C3—C4—C5	−0.62 (16)	C10—C11—C12—C13	1.41 (17)
C3—C4—C5—C6	−0.32 (18)	C11—C12—C13—C14	−0.32 (19)
C4—C5—C6—C1	0.93 (18)	C12—C13—C14—C9	−1.1 (2)
C6—C1—C2—C3	−0.32 (17)	C14—C9—C10—C11	−0.34 (19)
C7—O2—C3—C2	6.52 (16)	C15—O5—C11—C10	−9.16 (17)
C7—O2—C3—C4	−172.93 (11)	C15—O5—C11—C12	170.84 (11)
C8—O3—C4—C3	169.24 (12)	C16—O6—C12—C11	175.56 (13)
C8—O3—C4—C5	−9.38 (18)	C16—O6—C12—C13	−6.0 (2)

### Hydrogen-bond geometry (Å, º)

**Table:**

*D*—H···*A*	*D*—H	H···*A*	*D*···*A*	*D*—H···*A*
O1—H1···O2^i^	0.86 (1)	2.25 (1)	2.9663 (12)	141 (2)
O1—H1···O3^i^	0.86 (1)	2.13 (1)	2.8834 (13)	145 (2)
O4—H4···O5^i^	0.86 (1)	2.15 (2)	2.8384 (13)	137 (2)
O4—H4···O6^i^	0.86 (1)	2.37 (1)	3.1107 (14)	145 (2)

Symmetry code: (i) −*x*+1/2, *y*+1/2, *z*.

## References

[bb1] Bourhis, L. J., Dolomanov, O. V., Gildea, R. J., Howard, J. A. K. & Puschmann, H. (2015). *Acta Cryst.* A**71**, 59–75. 10.1107/S2053273314022207 PMC4283469 25537389

[bb2] Bruker (2014). *APEX2*, *SAINT*, and *SADABS*. Bruker AXS Inc., Madison, Wisconsin, USA.

[bb3] Dolomanov, O. V., Bourhis, L. J., Gildea, R. J., Howard, J. A. K. & Puschmann, H. (2009). *J. Appl. Cryst.* **42**, 339–341.

[bb4] Jia, X.-C., Li, J., Yu, Z.-R., Zhang, H. & Zhou, L. (2012). *Acta Cryst.* E**68**, o3160. 10.1107/S1600536812042997 PMC3515255 23284475

[bb5] McDonald, K. J., Desikan, V., Golen, J. A. & Manke, D. R. (2015). *Acta Cryst.* E**71**, o406. 10.1107/S2056989015009172 PMC4459336 26090192

[bb6] Nguyen, D. M., Desikan, V., Golen, J. A. & Manke, D. R. (2015). *Acta Cryst.* E**71**, o533. 10.1107/S2056989015012402 PMC4571382 26396782

[bb7] Sheldrick, G. M. (2008). *Acta Cryst.* A**64**, 112–122. 10.1107/S0108767307043930 18156677

[bb8] Sheldrick, G. M. (2015). *Acta Cryst.* C**71**, 3–8.

[bb9] Westrip, S. P. (2010). *J. Appl. Cryst.* **43**, 920–925.

[bb10] Yamamoto, H., Ohkubo, K., Akimoto, S., Fukuzumi, S. & Tsuda, A. (2014). *Org. Biomol. Chem.* **12**, 7004–7017. 10.1039/c4ob00659c 24947667

