# Supplementary figures and images for: Crystal structure of 3,4-di­meth­oxy­phenol
Source: Acta Crystallogr E Crystallogr Commun. 2015 Dec 6;71(Pt 12):o1019. doi: 10.1107/S2056989015022860 (PMC4719955; doi:10.1107/S2056989015022860)

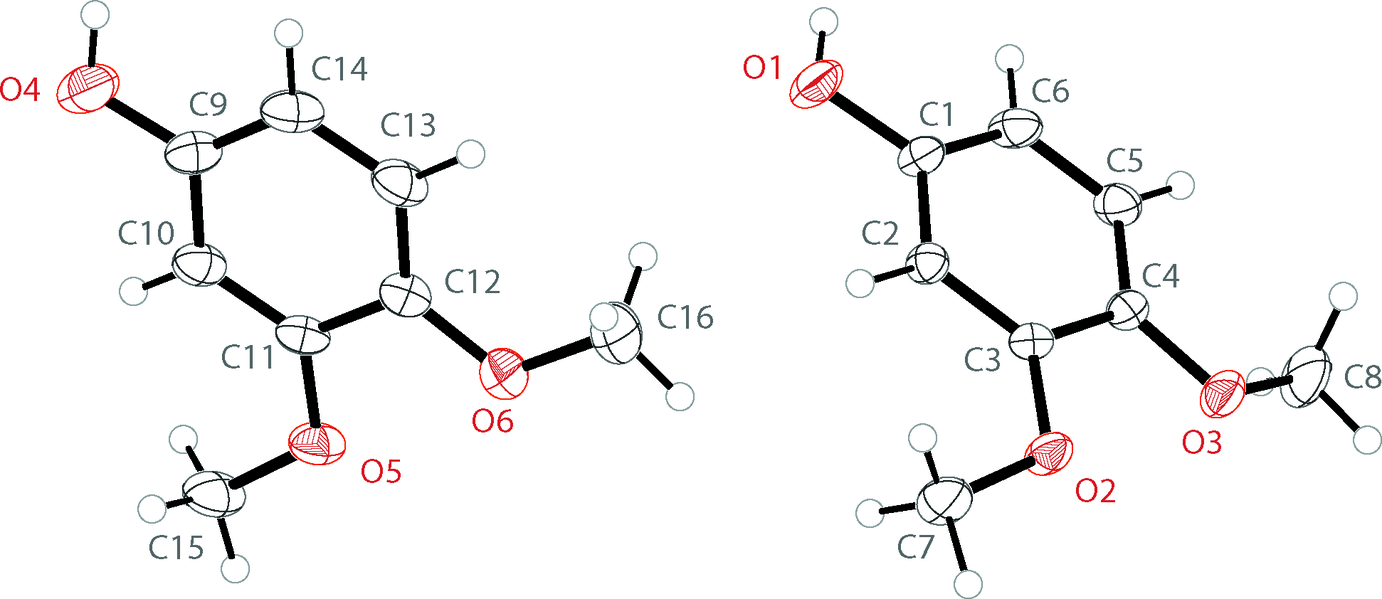

Supplement: Supplementary file 4 [file e-71-o1019-fig1.tif]

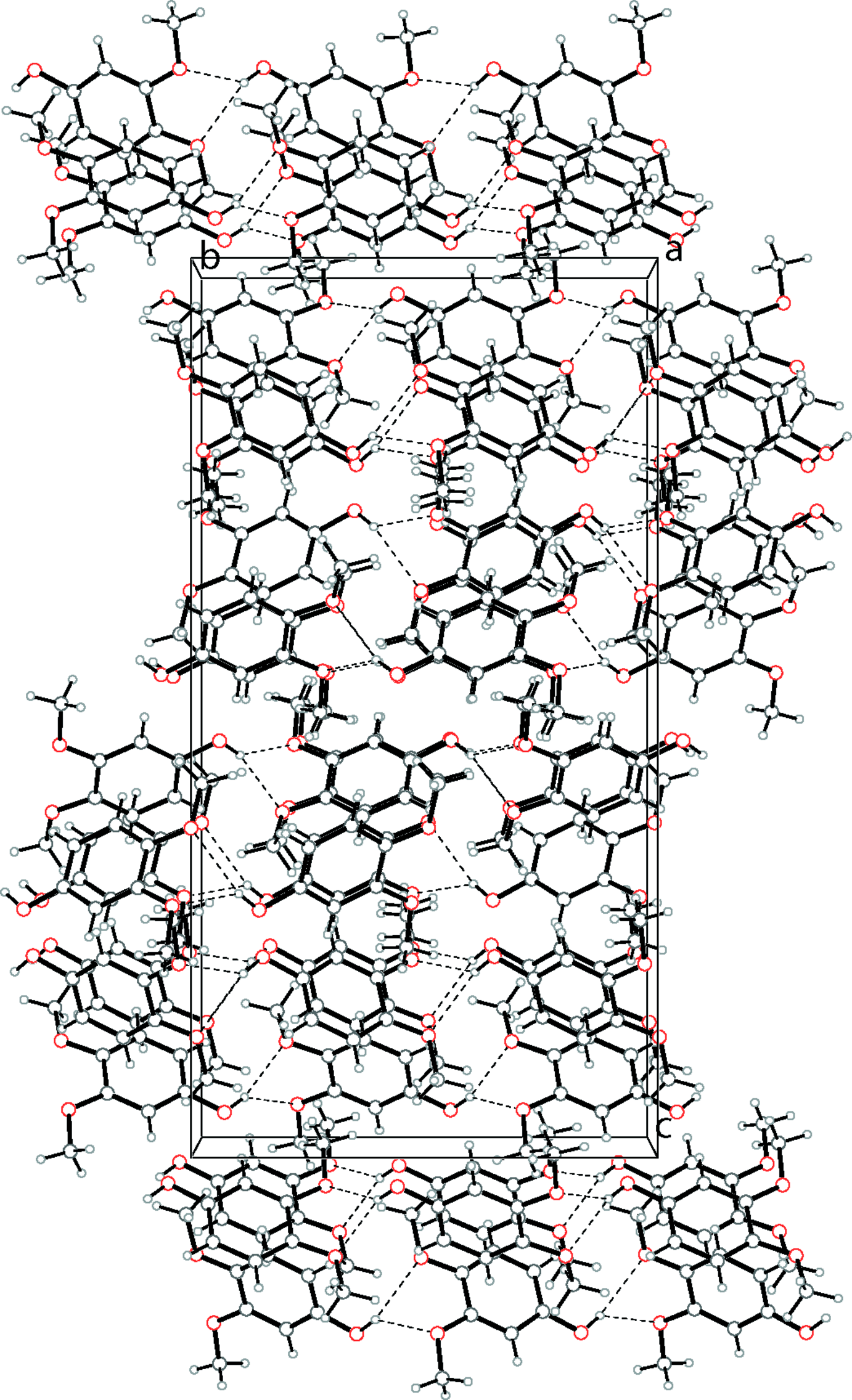

Supplement: Supplementary file 5 [file e-71-o1019-fig2.tif]
